# Effects of Statin Dose, Class, and Use Intensity on All-Cause Mortality in Patients with Type 2 Diabetes Mellitus

**DOI:** 10.3390/ph16040507

**Published:** 2023-03-29

**Authors:** Jung-Min Yu, Wan-Ming Chen, Mingchih Chen, Ben-Chang Shia, Szu-Yuan Wu

**Affiliations:** 1 Department of Cardiovascular Surgery, Taichung Tzu Chi Hospital, Taichung 427213, Taiwan; 2 Department of Surgery, School of Medicine, Tzu Chi University, Hualien 97004, Taiwan; 3 Graduate Institute of Business Administration, College of Management, Fu Jen Catholic University, Taipei 242062, Taiwan; 4 Artificial Intelligence Development Center, Fu Jen Catholic University, Taipei 242062, Taiwan; 5 Department of Food Nutrition and Health Biotechnology, College of Medical and Health Science, Asia University, Taichung 41354, Taiwan; 6 Division of Radiation Oncology, Lo-Hsu Medical Foundation, Lotung Poh-Ai Hospital, Yilan 265501, Taiwan; 7 Big Data Center, Lo-Hsu Medical Foundation, Lotung Poh-Ai Hospital, Yilan 265501, Taiwan; 8 Department of Healthcare Administration, College of Medical and Health Science, Asia University, Taichung 41354, Taiwan; 9 Cancer Center, Lo-Hsu Medical Foundation, Lotung Poh-Ai Hospital, Yilan 265501, Taiwan; 10 Centers for Regional Anesthesia and Pain Medicine, Taipei Municipal Wan Fang Hospital, Taipei Medical University, Taipei 11031, Taiwan; 11 Department of Management, College of Management, Fo Guang University, Yilan 26247, Taiwan

**Keywords:** type 2 diabetes, statin, dose-dependent, mortality, class of statin

## Abstract

Purpose: to examine the impact of statins on reducing all-cause mortality among individuals diagnosed with type 2 diabetes. This investigation explored the potential correlations between dosage, drug classification, and usage intensity with the observed outcomes. Methods: The research sample consisted of individuals aged 40 years or older diagnosed with type 2 diabetes. Statin usage was determined as a frequent usage over a minimum of one month subsequent to type 2 diabetes diagnosis, where the average statin dose was ≥28 cumulative defined daily doses per year (cDDD-year). The analysis employed an inverse probability of treatment-weighted Cox hazard model, utilizing statin usage status as a time-varying variable, to evaluate the impact of statin use on all-cause mortality. Results: The incidence of mortality was comparatively lower among the cohort of statin users (n = 50,804 (12.03%)), in contrast to nonusers (n = 118,765 (27.79%)). After adjustments, the hazard ratio (aHR; 95% confidence interval (CI)) for all-cause mortality was estimated to be 0.32 (0.31–0.33). Compared with nonusers, pitavastatin, rosuvastatin, pravastatin, simvastatin, atorvastatin, fluvastatin, and lovastatin users demonstrated significant reductions in all-cause mortality (aHRs (95% CIs) = 0.06 (0.04–0.09), 0.28 (0.27–0.29), 0.29 (0.28–0.31), 0.31 (0.30–0.32), 0.31 (0.30–0.32), 0.36 (0.35–0.38), and 0.48 (0.47–0.50), respectively). In Q1, Q2, Q3, and Q4 of cDDD-year, our multivariate analysis demonstrated significant reductions in all-cause mortality (aHRs (95% CIs) = 0.51 (0.5–0.52), 0.36 (0.35–0.37), 0.24 (0.23–0.25), and 0.13 (0.13–0.14), respectively; *p* for trend <0.0001). Because it had the lowest aHR (0.32), 0.86 DDD of statin was considered optimal. Conclusions: In patients diagnosed with type 2 diabetes, consistent utilization of statins (≥28 cumulative defined daily doses per year) was shown to have a beneficial effect on all-cause mortality. Moreover, the risk of all-cause mortality decreased as the cumulative defined daily dose per year of statin increased.

## 1. Introduction

Diabetes is a prominent contributor to global mortality rates and accounts for a position among the top 10 causes of death worldwide. In excess of 80% of premature deaths due to non-communicable diseases result from diabetes, cardiovascular disease, cancer, and respiratory disease collectively [1]. Type 2 diabetes affects a majority (over 90%) of the total number of individuals with diabetes worldwide and represents a significant health burden [2]. Type 2 diabetes is identified by hyperglycemia, insulin resistance, compromised insulin secretion, and dyslipidemia characterized by elevated triglyceride levels and reduced levels of high-density lipoprotein cholesterol [3,4,5,6]. Type 2 diabetes is associated with an elevated risk of heart disease, stroke, high blood pressure, atherosclerosis (narrowing of blood vessels), and peripheral neuropathy (nerve damage) [7,8]. The condition not only represents a significant risk factor for the aforementioned comorbidities, but it also increases the all-cause mortality risk by 35%, particularly in younger and female individuals [9]. However, there is a lack of research on the association between all-cause mortality, protective medication, and the relatively elderly (≥40 years old) type 2 diabetes population.

In patients with diabetes, the mortality rates are higher than in the general population; their prognosis following any cardiovascular event is generally worse as well [9,10,11]. The development of an effective protective medication against mortality in patients with type 2 diabetes is warranted and would be valuable. Statins, a commonly used medication, are often prescribed for patients with type 2 diabetes to help them manage their condition [12]. This is because type 2 diabetes increases the risk of heart disease, including heart attack and stroke [13]. Statin use does not indicate the failure of management of type 2 diabetes [12]. However, whether statin use in patients with type 2 diabetes reduces cardiovascular event incidence and all-cause mortality remains debatable [14,15,16,17,18,19,20,21,22,23,24,25,26,27,28,29]. Previous retrospective cohort studies have used vague and heterogeneous definitions of statin use: patients who used statins during hospitalization, had at least two invoices for statins during the enrolment period, or had statins listed on the medication list during the study period were considered statin users [26,27,28,29]. These definitions were not stratified by statin use dosage, statin class, or intensity (continuous or discontinuous use) [26,27,28,29]. Similarly, some randomized controlled trials (RCTs) have reported controversial conclusions [14,15,16,17,18,19,20,21,22,23,24,25] because they used a small sample size with heterogeneous endpoints and an insufficient follow-up duration; moreover, most of these RCTs did not state whether the study patients had type 1 or 2 diabetes [14,15,16,17,18,19,20,21,22,23,24,25].

Therefore, in the current study, we estimated the effects of statin use on the all-cause mortality of patients with type 2 diabetes and the dependency of these effects on the statin dose, class, and use intensity by using data from a real-world database. We also estimated the optimal daily statin dose of statins for patients with type 2 diabetes.

## 2. Results

Throughout the study period spanning from 2008 to 2020, a total of 849,787 patients were diagnosed with type 2 diabetes. The mean age at diagnosis was 56.85 years for nonusers and 56.92 years for users of statins. Atorvastatin was the most frequently prescribed statin (35.88%), followed by simvastatin (19.89%) and rosuvastatin (19.55%). To account for potential confounding factors, the IPTW Cox hazard model was used, resulting in balanced covariates between the two groups (Table 1).

### 2.1. Association of All-Cause Mortality with Different Statin Dosages and Classes

**Figure 1:**
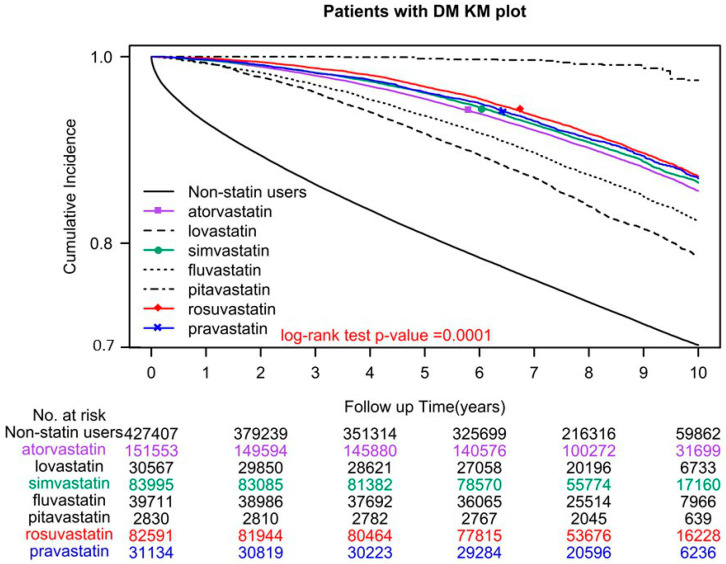
Kaplan–Meier overall survival curves of patients with type 2 diabetes who used different classes of statins.

A total of 118,765 (27.79%) individuals who did not use statins and 50,804 (12.03%) who did, died during the study period. The adjusted hazard ratio (aHR) for all-cause mortality was 0.32 (95% confidence interval (CI) = 0.31–0.33), indicating that statin users had lower mortality rates than nonusers (Table 2). Among statin users, users of pitavastatin, rosuvastatin, pravastatin, simvastatin, atorvastatin, fluvastatin, and lovastatin demonstrated a significant reduction in all-cause mortality, with aHRs (95% CIs) of 0.06 (0.04–0.09), 0.28 (0.27–0.29), 0.29 (0.28–0.31), 0.31 (0.30–0.32), 0.31 (0.30–0.32), 0.36 (0.35–0.38), and 0.48 (0.47–0.50), respectively (Table 2). In the log-rank test, overall survival was significantly different for different statin classes used (*p* < 0.0001; Figure 1).

**Figure 2:**
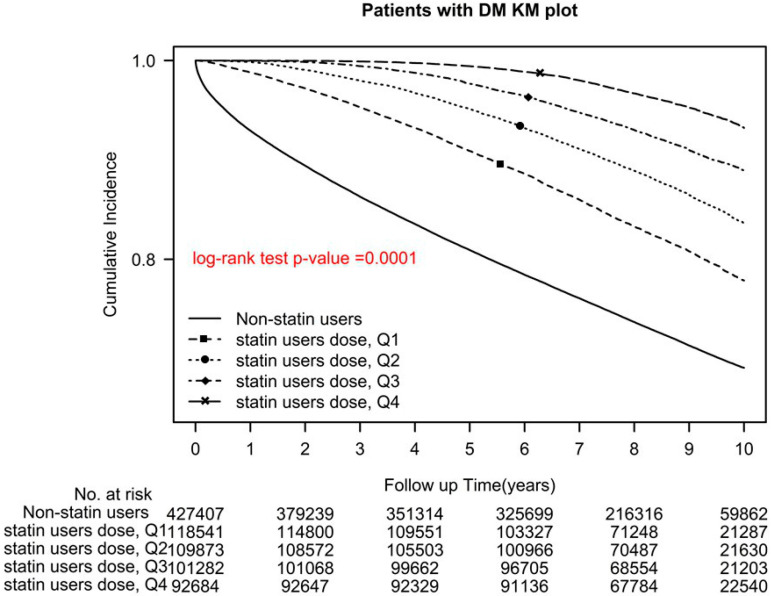
Kaplan–Meier overall survival curves of patients with type 2 diabetes who used different cDDD-year of statins.

Among statin users, users of Q1, Q2, Q3, and Q4 cDDD-year of statins demonstrated a significant reduction in all-cause mortality, with aHRs (95% CIs) of 0.51 (0.5–0.52), 0.36 (0.35–0.37), 0.24 (0.23–0.25), 0.13 (0.13–0.14), respectively (*p* for trend < 0.0001; *p* < 0.0001, log-rank test; Figure 2).

### 2.2. Statin Use Intensity

**Figure 3:**
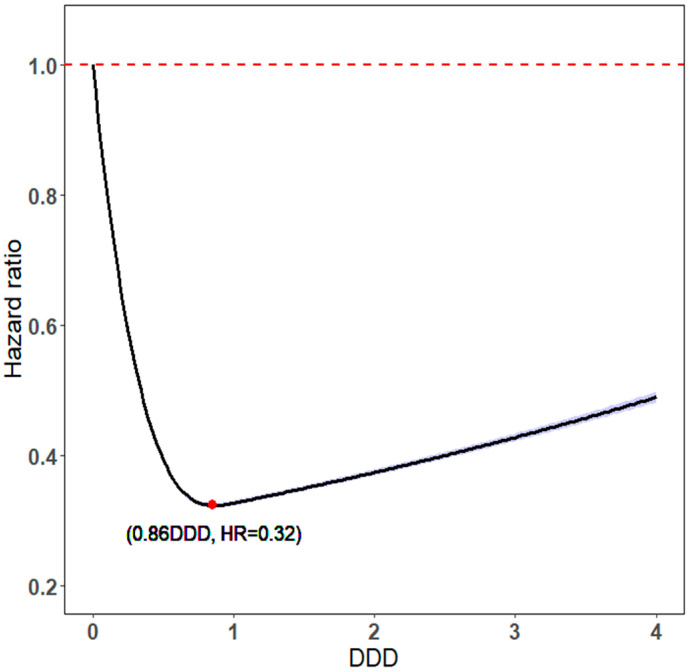
DDD of statin use vs. HRs for all-cause mortality.

The optimal statin dose was 0.86 DDD, with the lowest aHR of 0.32 (Figure 3). The protective effects on mortality and dose–response relationships exhibited U-shaped dose–response relationships [30], which means a higher DDD is not always associated with a lower risk of mortality.

### 2.3. Sensitivity Analysis

We conducted a sensitivity analysis that involved patients who initiated statins either after or before the diagnosis of type 2 diabetes, and the results indicated that statin use was linked with a reduction in all-cause mortality in both groups (Table 3). We also investigated the influence of statin use intensity and found that mortality decreased in patients who used an average of ≤1 and >1 DDD. Additionally, we examined the effects of statins in patients with different comorbidities (CCI ≤ 1), age groups, sexes, income levels, urbanization levels, numbers of antidiabetic drug types used, antidiabetic drugs used, aDCSI scores, and new or prevalent statin use. The reductions in all-cause mortality observed in the sensitivity analysis were similar to those obtained in the primary analysis (Table 3).

## 3. Discussion

This study presents novel findings on the dose-dependent effects, specific class, and intensity of statin use on all-cause mortality in patients with type 2 diabetes. This study is the leading study to provide real-world evidence showing that persistent statin use, particularly at higher cumulative doses per year, is associated with reduced all-cause mortality in these patients. The study also identifies the optimal daily dose of statins as 0.86 DDD, which is associated with the lowest mortality. Additionally, the study ranks the priority of protective effects on mortality for different classes of statins, with pitavastatin demonstrating the highest protective effects, followed by rosuvastatin, pravastatin, simvastatin, atorvastatin, fluvastatin, and lovastatin. These novel findings clarify the protective effects of dose-dependence and intensity on statin users and specific classes of statin use on mortality in patients with type 2 diabetes, which has not been previously investigated [14,15,16,17,18,19,20,21,22,23,24,25,26,27,28,29].

A recent meta-analysis showed that statin use significantly reduced the risk of CVD events and stroke, but not all-cause mortality, in individuals with diabetes undergoing both primary and secondary prevention [31]. The outcomes seemed different from ours. The potential reasons might be that our study focused on the association between statin use and all-cause mortality specifically in individuals with type 2 diabetes. In contrast, Yang XH et al.’s meta-analysis assessed the effect of statin use on a broader range of outcomes, including heterogeneous endpoints such as CVD events and stroke, which were different primary endpoints. Furthermore, the meta-analysis used a heterogeneous study design, including RCTs, observational cohort studies, and retrospective studies. The meta-analysis also included a population that was not solely comprised of individuals with type 2 diabetes, which limited the extrapolation of results to this specific patient population. In addition, our study used a different methodology, which was very large and adjusted for potential confounding factors using IPTW Cox regression models, whereas the meta-analysis may have used different statistical techniques. The meta-analysis did not provide data on the dose, intensity, or class of statin use, whereas our study presented novel findings on the dose-dependent effects, specific class, and intensity of statin use on all-cause mortality in patients with type 2 diabetes.

Numerous studies, both observational and randomized controlled trials (RCTs), have suggested that there is a correlation between the use of statins and a decrease in all-cause mortality in individuals with diabetes [14,15,16,17,18,19,20,21,22,23,24,25,26,27,28,29]. The results of these studies are debatable because they did not clarify the statin dosage, intensity, or classes used; moreover, they used a small sample size with heterogeneous endpoints and an insufficient follow-up duration and did not classify patients based on their diabetes type [26,27,28,29]. The present study is the first to verify the preventive properties of various classes and use intensities of statins against all-cause mortality in patients diagnosed with type 2 diabetes. We used an IPTW design to estimate the long-term overall survival of patients using specific statin classes at different dosages (cDDD-year) and intensities (>1 or ≤1 DDD); we also estimated the optimal daily dose (DDD) of statin for type 2 diabetes. Our results demonstrated a significant reduction in all-cause mortality among pitavastatin, rosuvastatin, pravastatin, simvastatin, atorvastatin, fluvastatin, and lovastatin users. Moreover, a significant reduction was noted in all-cause mortality among users of Q1, Q2, Q3, and Q4 cDDD-year of statin. Regardless of age, sex, income level, urbanization level, number of antidiabetic drugs used, type of antidiabetic drug used, aDCSI score, comorbidities, and CCI score, statin use at ≥28 cDDD-year significantly reduced all-cause mortality. Compared with no statin use, pitavastatin had the highest protective effects against mortality, followed by rosuvastatin, pravastatin, simvastatin, atorvastatin, fluvastatin, and finally, lovastatin. Moreover, the optimal statin dose was noted to be 0.86 DDD, which was associated with the lowest mortality.

To date, no study has compared the impact of different statin classes on all-cause mortality in individuals with type 2 diabetes. The current study is the first to demonstrate the order of intensity by which specific statin classes affect mortality in patients with type 2 diabetes: pitavastatin > rosuvastatin > pravastatin > simvastatin > atorvastatin > fluvastatin > lovastatin. The mechanisms underlying this order may be associated with the effects of each statin on high-density lipoprotein (HDL), low-density lipoprotein (LDL), and triglycerides [32,33,34]. For instance, rosuvastatin is slightly more potent than atorvastatin [32,33]; it is also significantly more potent than pravastatin, simvastatin, atorvastatin, fluvastatin, and lovastatin [33,34]. At maximal prescribed doses, rosuvastatin provides a greater LDL reduction than other statins [33,34]. Statin therapy alters HDL levels, typically by increasing them. However, these effects may vary by the statin type and dose [35]. For instance, simvastatin and rosuvastatin increase HDL levels with an increase in the dose, whereas an increase in HDL levels is noted at a high dose of atorvastatin [35]. Moreover, in patients with hypercholesterolemia, rosuvastatin is more effective at decreasing triglycerides than are other statins [33]. The magnitude of the triglyceride-decreasing effect of statins may be as high as 40%–44% in patients with hypertriglyceridemia [32,33,34,35]. However, the association of specific statins’ potency and effects on LDL, HDL, and triglycerides with mortality remains unclear. In the current study, this appeared be in proportion with the order of intensity of the statins’ effects in patients with type 2 diabetes (Table 2 and Figure 1). Moreover, certain statins, such as fluvastatin, pitavastatin, and pravastatin, are associated with a lower risk of drug interactions and muscle toxicity compared to other statins. For example, pravastatin, fluvastatin, rosuvastatin, and pitavastatin do not undergo CYP3A4 metabolism; therefore, fewer pharmacokinetic drug interactions are expected with these agents [36,37]. In general, patients with type 2 diabetes tend to use different types of medications (Table 1); therefore, statins, such as pitavastatin, demonstrating few drug–drug interactions, might be preferable [36,37]. Although the underlying mechanisms remain unclear, statins with fewer drug–drug interactions, such as pitavastatin [36,37], or those with stronger LDL and triglyceride-lowering and HDL-increasing effects, such as rosuvastatin [32,33,34], might be ideal for use in patients with type 2 diabetes. However, the sample size of pitavastatin users in our study was small; therefore, the current conclusion might be biased, and further research is warranted.

The intensity and daily dose of statin use is complicated by LDL, HDL, and triglycerides because the protective effects of DDD on LDL, HDL, and triglycerides exhibit U-shaped dose–response relationships (Figure 3) [35,38]. Thus, the U-shaped dose–response relationship has been noted for not only the pharmacological effects but also the toxicologic effects of statins on mortality (Figure 3) [30]; this relationship was also noted in the current study: the higher the daily statin dose, the higher the protective effect [39]. In the current study, the optimal DDD was 0.86 for statin users because it was associated with the lowest all-cause mortality, a result compatible with the U-shaped dose–response relationship noted in previous biological, toxicological, and pharmacological studies [30]. Individual variability in the response to and side effects of statins may be related to differences in drug metabolism rates that stem from genetic variations [40,41,42]. For instance, certain genetic differences such as the absence of CYP2D6, a member of the cytochrome P450 superfamily of drug oxidizing enzymes, in 7% of Caucasian and African–American individuals, can impact drug metabolism rates, whereas CYP2D6 deficiency is rare among Asian individuals. Asian (mostly Chinese, Japanese, and Korean) individuals may have a higher response to low statin doses than do Caucasian individuals [41]. In Asian individuals, the initial daily dose of statins should ideally be lower than that in individuals of other ethnicities [41,43]; this is corroborated by the optimal statin DDD noted in the current study.

We investigated the potential impact of different cumulative doses of continuous, discontinuous, or cDDD-year statin use on LDL, HDL, and triglycerides, as well as their effects on all-cause mortality in patients with type 2 diabetes. The analysis revealed that a higher cDDD-year of statin usage corresponded with a lower all-cause mortality in this patient population. Additionally, we explored the influence of specific levels of statin dosage, namely >1 and ≤1 DDD, and found that both levels of use resulted in a significant reduction in all-cause mortality, with ≤1 DDD demonstrating a higher reduction than >1 DDD. These findings may align with the U-shaped relationship previously established between statin effects and LDL [30,38].

The paper from Scicchitano P et al. (2014) highlights the potential role of nutraceuticals in improving dyslipidemia, a major cardiovascular risk factor for coronary heart disease [44]. The authors suggest that nutraceuticals and functional food ingredients may be useful in reducing overall cardiovascular risk induced by dyslipidemia, acting either parallel to statins or as adjuvants in cases where statins cannot be used or fail. The potential mechanisms by which nutraceuticals may act on lipids include reducing 7α-hydroxylase, increasing the fecal excretion of cholesterol, decreasing 3-hydroxy-3-methylglutaryl-CoA reductase mRNA levels, or reducing the secretion of very low-density lipoprotein. However, the exact mechanisms are not yet fully understood. While nutraceuticals may have potential benefits in improving dyslipidemia, the use of these compounds in type 2 diabetes patients is not paid by the National Health Insurance. Moreover, the effects of nutraceuticals on the primary endpoint of all-cause mortality in type 2 diabetes patients are still controversial, and it is unclear whether nutraceutical use is a confounding factor in determining all-cause mortality in type 2 diabetes patients. Therefore, while the potential role of nutraceuticals in improving dyslipidemia is promising, more research is needed to fully understand their effects on type 2 diabetes patients, particularly in relation to mortality outcomes. In the context of this study, the effects of nutraceuticals on the primary endpoint of all-cause mortality in type 2 diabetes patients were not examined, and their potential influence on the results of the study cannot be fully assessed.

It is important to note that in the real-world database used for this study, all type 2 diabetes patients receive treatments based on the professional physicians who prescribe medications for the patients according to diabetes guidelines and are monitored by peer reviewers in Taiwan. If the prescriptions are found to be against the regulations and coverage of NHI, then physicians face punishment and are not paid. Therefore, it is difficult to analyze all pharmacological compounds in the real-world database as not all drugs are covered by Taiwan NHI. However, all antidiabetic drugs were considered and adjusted in the type 2 diabetes population to achieve balance between the case and control groups. After PSM, only statin use was found to be different between the case and control groups (Table 1). While it would be ideal to include all pharmacological compounds in the analysis, it was not feasible in this study due to the limitations of the real-world database. Nevertheless, the effect of statin use on all-cause mortality in type 2 diabetes patients has been well established in previous studies and was included in the multivariate regression analysis.

**Figure 4:**
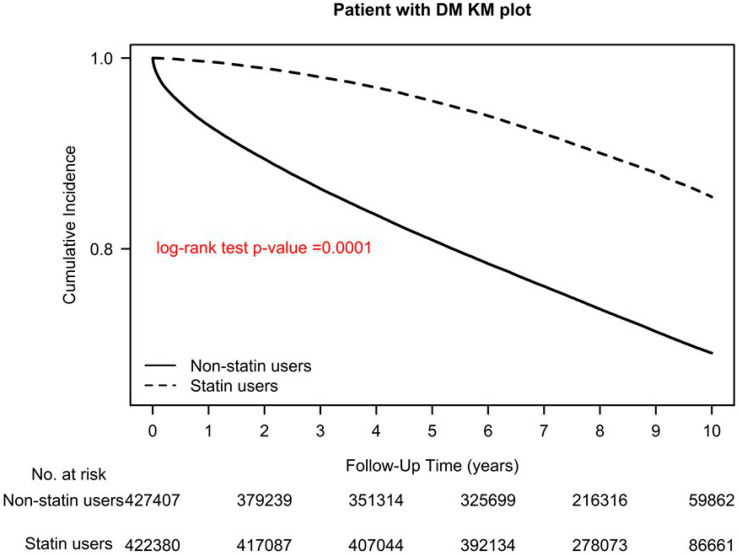
Kaplan–Meier overall survival curves of patients with type 2 diabetes who used and did not use statins.

The main strength of the current study is the large sample size. We also considered the intensity of statin use (>1 DDD (continuous) or ≤1 DDD (discontinuous)) and analyzed it by using a sensitivity analysis, and it was adjusted using a Cox hazard model. Regardless of statin use intensity, statin users had decreased all-cause mortality compared with nonusers. In contrast to the previous relevant studies [14,15,16,17,18,19,20,21,22,23,24,25,26,27,28,29], our study obtained reliable real-world evidence through long-term follow-up, which demonstrated that persistent statin use reduces all-cause mortality in patients with type 2 diabetes (Figure 1, Figure 2 and Figure 4). We also noted that the optimal daily statin dose was 0.86 DDD (Figure 2). Moreover, pitavastatin demonstrated the most protective effect, followed by rosuvastatin, pravastatin, simvastatin, atorvastatin, fluvastatin, and lovastatin (Table 2 and Figure 1).

This study has several limitations. Firstly, the data were obtained from a claims database, which means that we could not collect detailed information such as the blood and lipid profiles of each patient, and thus, we could not evaluate whether changes in lipid profiles after initiating statin treatment were associated with mortality. Secondly, we could not eliminate the possibility of selection bias due to unmeasured confounders, as statin users may differ from nonusers. To address this, we used an IPTW Cox hazard model to balance the differences in the covariates and conducted subgroup analyses. We found that the reductions in mortality with statin use were consistent across various subgroups. Thirdly, we were unable to collect information on the body mass index, dietary information, and other lifestyle factors at the time of type 2 diabetes diagnosis. Fourthly, it is possible that the study’s findings may not be generalizable to frail individuals who may not attend regular health check-ups or who may not be prescribed statins due to their frailty. Fifth, small event numbers in some subgroups that used a single type of statin limited the statistical power of our results. Sixth, we could not analyze the use of self-pay nutraceuticals, which are not covered by the NHI. However, the effects of nutraceuticals on all-cause mortality in type 2 diabetes patients remain controversial, and their use as a confounding factor for all-cause mortality is still unclear. Finally, we relied on a sample population that was 95% Han Chinese, which may not be entirely generalizable to other ethnic groups [45]. It is worth noting that the prevalence of statin use varies by region, with usage rates of approximately 76.5%, 69.9%, and 60.5% in North America, western Europe, and Asia, respectively [46]. As a result, populations of other ethnicities with high rates of statin use may yield slightly different outcomes than our results suggest. Nevertheless, other studies conducted in various ethnic populations have indicated a decrease in the risk of mortality related to statin use [14,15,16,17,18,19,20,21,22,23,24,25,26,27,28,29].

## 4. Methods

### 4.1. Study Population

A population-based cohort study was carried out utilizing the Taiwan National Health Insurance (NHI) Research Database (NHIRD). All beneficiaries’ medical claims data pertaining to disease diagnoses, procedures, drug prescriptions, demographics, and enrollment profiles are included in the NHIRD [47]. The NHIRD data are linked by encrypted patient identifiers, and it also includes the vital status and cause of death of each patient, extracted from Taiwan’s death registry.

The cohort enrolled in our study consisted solely of patients aged ≥40 years who had been diagnosed with type 2 diabetes between 2008 and 2020. Patients with missing data on the age at diagnosis or date of diagnosis were excluded. Moreover, we excluded patients who used multiple classes of statins during the follow-up period. The index date was the date of statin use (≥28 cDDD-year). The observation period for each patient began from the index date and continued until death, or the end of the study period (31 December 2021). Patients with T2DM who were prescribed ≥28 cDDD-year of statins with a prescription duration of >1 months were included in the case group, and those who were prescribed 0 cDDD of statins during the follow-up period were included in the control group. The Institutional Review Board of Tzu-Chi Medical Foundation reviewed and granted approval of the study protocols (IRB109-015-B).

### 4.2. Study Covariates

We included other covariates to adjust for potential confounding effects. Patients were divided into the following age groups: 40 to 50, 51 to 60, 61 to 70, and ≥71 years at the index date. To reduce the effects of potential confounders when comparing all-cause mortality between the statin user and nonuser groups, we used the inverse probability of treatment-weighted (IPTW) [48]. We used the date of statin use (≥28 cDDD-year) as the index date and matched statin nonusers by using variables collected at this index date. The factors included age, sex, income level, urbanization level, number of antidiabetic drug types used, antidiabetic drugs used, diabetes severity (based on adapted Diabetes Complications Severity Index score), and comorbidities, which were determined based on International Classification of Diseases codes. Comorbidity onsets over one year before the index date were recorded. Continuous variables are presented as means ± standard deviations or medians (first quartile, third quartile) where appropriate. Charlson’s comorbidity index (CCI) score was also calculated, with repeat comorbidities excluded to avoid repetitive adjustments in multivariate analysis. The flowchart depicting the study selection process is presented as Supplemental Figure S1.

### 4.3. Outcome Variables

The primary variable of interest in this study was mortality due to any cause, which was identified using information from the death registry after the diagnosis of type 2 diabetes.

### 4.4. Statin Use

Pharmaceutical claims data on statin prescriptions were retrieved using Anatomical Therapeutic Chemical (ATC) codes from the NHIRD. To examine the major exposures of interest, lipophilic (atorvastatin, fluvastatin, lovastatin, simvastatin, and pitavastatin) and hydrophilic (pravastatin and rosuvastatin) statins were selected based on the ATC classification system [49]. Data on statin use initiated 1 year prior to type 2 diabetes diagnosis were extracted to differentiate prevalent and new users. We also evaluated statin use intensity by estimating the average statin dose as the defined daily dose (DDD) divided by the total prescription days. Statin use intensity was categorized into two groups: average daily doses below or above 1 DDD. Additionally, patients were divided into four subgroups based on quartiles (Qs) of cDDD-year. All analyses were adjusted for covariates, including age group, sex, income level, urbanization level, number of antidiabetic drug types used, antidiabetic drugs used, aDCSI score, comorbidities, and CCI score to reduce potential confounding effects on the outcome variable of all-cause mortality, as determined by the cause of death data in the death registry after type 2 diabetes diagnosis.

### 4.5. Statistical Analysis

A time-dependent Cox hazard model was utilized to evaluate overall survival in relation to statin use, adjusted for age group, sex, income level, urbanization level, number of antidiabetic drug types used, antidiabetic drugs used, aDCSI score, comorbidities, and CCI score. Statin prescriptions were collected every 3 months as a time-dependent variable to determine a user’s status, with “event-free” person-times of users before their first statin prescription and during the 3-month period without a statin prescription considered unexposed follow-up time points. Overall survival risk was also estimated for individual statins. Subgroup analyses, adjusted for baseline characteristics, were performed using stratification instead of weighting and postdiagnosis statin use, with similar results obtained. All-cause mortality was estimated using the Kaplan–Meier method, and the stratified log-rank test was employed to compare survival curves between statin users and nonusers (Figure 4), and between nonusers and statin users using different statin dosages and classes (Figure 1 and Figure 2). SAS (version 9.4; SAS Institute, Cary, NC, USA) was used for all statistical analyses.

## 5. Conclusions

In conclusion, our real-world evidence indicated that persistent statin use (≥28 cDDD-year) may reduce all-cause mortality in patients with type 2 diabetes: the higher the cDDD-year of statin use, the lower the all-cause mortality. The optimal daily statin dose, which led to the lowest all-cause mortality, was 0.86 DDD. Moreover, the protective effect against mortality was the highest in with the use of pitavastatin, followed by rosuvastatin, pravastatin, simvastatin, atorvastatin, fluvastatin, and, finally, lovastatin.

## Figures and Tables

**Table 1:** Baseline characteristics of type 2 diabetes cohort: overall and stratified by statin use.

	Nonusers		Users		*p*	ASMD
	*N* = 427,407		*N* = 422,380			
Characteristic	n	%	n	%		
**Age**, mean ± SD, years	56.85 ± 20.97		56.92 ± 19.24		0.8520	
**Age**, median (IQR), years	56.00 (46.00, 68.00)		56.00 (48.00, 68.00)		0.9999	
**Age group**, years					0.0844	0.0046
≤50	143,911	33.67%	141,194	33.43%		
51–60	112,251	26.26%	111,046	26.29%		
61–70	86,430	20.22%	86,057	20.37%		
≥71	84,815	19.84%	84,083	19.91%		
**Sex**					0.6946	0.0004
Female	202,041	47.27%	199,485	47.23%		
Male	225,366	52.73%	222,895	52.77%		
**Income levels (NTD)**					0.6213	0.0008
Low income	6860	1.61%	6702	1.59%		
Financially dependent	135,057	31.60%	133,548	31.62%		
≤20,000	202,250	47.32%	200,462	47.46%		
20,001–30,000	38,833	9.09%	38,088	9.02%		
30,001–45,000	28,027	6.56%	27,510	6.51%		
>45,000	16,380	3.83%	16,070	3.80%		
**Urbanization**					0.9444	0.0001
Rural	121,995	28.54%	120,589	28.55%		
Urban	305,412	71.46%	301,791	71.45%		
**Number of antidiabetic drug types used**					0.0701	0.0009
0	156,611	36.64%	155,804	36.89%		
1	105,742	24.74%	104,725	24.79%		
2	105,362	24.65%	103,280	24.45%		
3	43,350	10.14%	42,551	10.07%		
≥4	16,342	3.82%	16,020	3.79%		
**Antidiabetic drugs used**						
Insulin	45,219	10.58%	44,743	10.59%	0.9485	0.0002
Metformin	183,186	43.86%	181,487	42.97%	0.5920	0.0007
SU	206,950	48.42%	204,777	48.48%	0.3972	0.0006
AGI	3479	0.81%	3473	0.82%	0.4462	0.0001
TZD	27,054	6.33%	26,950	6.38%	0.6642	0.0002
DPP4i	21,071	4.93%	20,903	4.95%	0.7950	0.0002
SGLT2i	488	0.11%	464	0.11%	0.9429	0.0001
Others	24,661	5.78%	24,412	5.78%	0.9652	0.0001
**aDCSI score**						
Mean ± SD	1.00 ± 1.89		1.03 ± 1.44		0.5461	
Median (IQR)	0.00 (0.00, 2.00)		0.00 (0.00, 2.00)		0.5659	
**aDCSI score**					0.7967	0.0059
0	219,618	51.38%	217,419	51.47%		
1	89,009	20.83%	87,662	20.75%		
2	65,173	15.25%	64,273	15.22%		
≥3	53,607	12.54%	53,026	12.55%		
Retinopathy	24,661	5.77%	24,395	5.78%	0.8936	0.0004
Nephropathy	50,647	11.85%	50,118	11.87%	0.8851	0.0002
Neuropathy	44,450	10.40%	44,130	10.45%	0.3064	0.0008
Cerebrovascular	45,518	10.65%	45,226	10.71%	0.7408	0.0002
Cardiovascular	113,946	26.66%	113,559	26.89%	0.8863	0.0002
Peripheral vascular disease	16,113	3.77%	15,914	3.77%	0.9940	0.0001
Metabolic disorder	7738	1.81%	7734	1.83%	0.8046	0.0001
**Comorbidities**						
Hypertension	219,833	51.43%	217,360	51.46%	0.8053	0.0003
Coronary artery disease	96,754	22.64%	95,261	22.55%	0.3541	0.0008
Stroke	62,388	14.60%	61,602	14.58%	0.8697	0.0001
Depression	28,112	6.58%	28,035	6.64%	0.2645	0.0006
Anxiety	59,006	13.81%	58,624	13.88%	0.3245	0.0007
Heart failure	28,686	6.71%	28,508	6.75%	0.4897	0.0004
Peripheral vascular disease	9221	2.16%	9091	2.15%	0.8691	0.0001
COPD	88,209	20.64%	86,839	20.56%	0.3698	0.0008
Atrial fibrillation	9495	2.22%	9328	2.21%	0.6841	0.0001
Traumatic head injury	26,003	6.08%	25,696	6.08%	0.9955	0.0000
Hearing loss	11,359	2.66%	11,365	2.69%	0.3464	0.0003
Sleep apnea	2423	0.57%	2349	0.56%	0.5036	0.0001
Liver cirrhosis	119,973	28.07%	118,674	28.10%	0.2204	0.0023
SLE	6592	1.54%	6547	1.55%	0.7749	0.0001
**CCI scores**						
Mean ± SD	1.10 ± 2.10		1.20 ± 1.58		0.1397	
Median (Q1, Q3)	0.00 (0.00, 2.00)		1.00 (0.00, 2.00)		0.9628	
**CCI scores**					0.0785	0.0019
0	229,905	53.79%	226,397	53.60%		
≥1	197,503	46.21%	195,983	46.40%		
**Different classes of statins**						
**Lipophilic statins**						
Atorvastatin	0	0.00%	151,553	35.88%		
Lovastatin	0	0.00%	30,567	7.24%		
Simvastatin	0	0.00%	83,995	19.89%		
Fluvastatin	0	0.00%	39,711	9.40%		
Pitavastatin	0	0.00%	2830	0.67%		
**Hydrophilic statins**						
Rosuvastatin	0	0.00%	82,591	19.55%		
Pravastatin	0	0.00%	31,134	7.37%		
**cDDD-year of statins**						
Q1	0	0.00%	118,541	28.06%		
Q2	0	0.00%	109,873	26.01%		
Q3	0	0.00%	101,282	23.98%		
Q4	0	0.00%	98,684	21.94%		
**DDD**						
≤1	0	0.00%	143,141	33.89%		
>1	0	0.00%	279,239	66.11%		
**Stain use**						
New use (after type 2 diabetes diagnosis)	0	0.00%	384,108	90.94%		
Prevalent use (before type 2 diabetes diagnosis)	0	0.00%	38,272	9.06%		
**Time from type 2 diabetes diagnosis to statins exposure**						
Mean ±SD follow-up			2.42 ± 2.69			
Median (IQR) follow-up			1.33 (0.07, 4.19)			
**Follow-up duration**						
Mean ± SD follow-up	8.04 ± 3.12		9.48 ± 1.76		<0.0001	
Median (IQR) follow-up	8.97 (5.66, 9.33)		9.65 (7.58, 9.76)		<0.0001	
**All-cause mortality**					<0.0001	
No	308,643	72.21%	371,576	87.97%		
Yes	118,765	27.79%	50,804	12.03%		

**Abbreviations**: ASMD, absolute standardized mean difference; SD, standard deviation; IQR, interquartile range; Q, quartile; DDD, defined daily dose; AIDS, acquired immunodeficiency syndrome; CCI, Charlson’s comorbidity index; COPD, chronic obstructive pulmonary disease; SLE, systemic lupus erythematosus; NTD, New Taiwan Dollar; aDCSI, adapted Diabetic Complication Severity Index; SU, sulfonylureas; AGI, alpha-glucosidase inhibitor; TZD, thiazolidinedione; DPP4i, dipeptidyl peptidase 4 inhibitor; SGLT2i, sodium–glucose cotransporter-2 inhibitor.

**Table 2:** All-cause mortality and aHRs for statin use in patients with type 2 diabetes.

Variables	Crude HR (95%CI)		*p*	aHR (95%CI) *		*p*
**Stain user or nonusers**						
Nonusers	**Reference**					
Users	0.37	(0.36, 0.37)	<0.0001	0.32	(0.31, 0.33)	<0.0001
**Different classes of statins**						
Nonusers	**Reference**					
**Hydrophilic statins**						
Rosuvastatin	0.32	(0.31, 0.34)	<0.0001	0.29	(0.28, 0.31)	<0.0001
Pravastatin	0.31	(0.3, 0.32)	<0.0001	0.28	(0.27, 0.29)	<0.0001
**Lipophilic statins**						
Atorvastatin	0.05	(0.03, 0.07)	<0.0001	0.06	(0.04, 0.09)	<0.0001
Lovastatin	0.47	(0.45, 0.48)	<0.0001	0.36	(0.35, 0.38)	<0.0001
Simvastatin	0.34	(0.33, 0.35)	<0.0001	0.31	(0.30, 0.32)	<0.0001
Fluvastatin	0.58	(0.56, 0.61)	<0.0001	0.48	(0.47, 0.50)	<0.0001
Pitavastatin	0.36	(0.36, 0.37)	<0.0001	0.31	(0.31, 0.32)	<0.0001
**cDDD-year of statins**						
Nonusers	**Reference**					
Q1	0.61	(0.6, 0.62)	<0.0001	0.51	(0.5, 0.52)	<0.0001
Q2	0.41	(0.4, 0.42)	<0.0001	0.36	(0.35, 0.37)	<0.0001
Q3	0.27	(0.26, 0.27)	<0.0001	0.24	(0.23, 0.25)	<0.0001
Q4	0.15	(0.14, 0.15)	<0.0001	0.13	(0.13, 0.14)	<0.0001
*p* for trend			<0.0001			<0.0001

**Abbreviations**: aHR, adjusted hazard ratio; HR, hazard ratio, CI, confidence interval; DDD, defined daily dose; Q, quartile. * aHR was derived from the inverse probability treatment-weighted Cox model considering statin use as a time-dependent covariate and was adjusted for age group, sex, income level, urbanization, antidiabetic drug type, antidiabetic drug use, aDCSI score, comorbidities, and CCI score.

**Table 3:** Sensitivity analyses for statin use–all-cause mortality association in patients with type 2 diabetes.

Subpopulation or Exposure	No. of Patients	All-Cause Mortality			
		No. of Deaths	aHR *	95% CI	*p*
**Age group**, years					
≤50	285,105	23,316	0.29	(0.28–0.30)	<0.0001
51–60	223,297	27,319	0.31	(0.30–0.32)	<0.0001
61–70	172,487	37,672	0.33	(0.32–0.34)	<0.0001
≥71	168,898	81,260	0.32	(0.32–0.33)	<0.0001
**Sex**					
Female	401,526	68,131	0.3	(0.30–0.31)	<0.0001
Male	448,261	101,438	0.33	(0.33–0.34)	<0.0001
**Income levels (NTD)**					
Low income	13,562	4936	0.35	(0.32–0.38)	<0.0001
Financially dependent	268,604	62,198	0.33	(0.32–0.33)	<0.0001
≤20,000	402,713	90,946	0.31	(0.31–0.32)	<0.0001
20,001–30,000	76,921	5847	0.34	(0.31–0.36)	<0.0001
30,001–45,000	55,537	3713	0.32	(0.29–0.35)	<0.0001
>45,000	32,450	1928	0.41	(0.36–0.47)	<0.0001
**Urbanization**					
Rural	242,584	58,568	0.31	(0.30–0.32)	<0.0001
Urban	607,203	111,001	0.33	(0.32–0.33)	<0.0001
**Number of antidiabetic drug types used**					
0	312,415	50,615	0.33	(0.32–0.34)	<0.0001
1	210,467	43,730	0.30	(0.29–0.31)	<0.0001
2	208,642	40,260	0.33	(0.32–0.34)	<0.0001
3	85,901	24,499	0.31	(0.30–0.32)	<0.0001
≥4	32,362	10,464	0.33	(0.31–0.35)	<0.0001
**aDCSI score**					
0	437,037	53,522	0.31	(0.31–0.32)	<0.0001
1	176,671	27,167	0.36	(0.34–0.37)	<0.0001
2	129,446	39,528	0.29	(0.28–0.30)	<0.0001
≥3	106,633	49,352	0.33	(0.32–0.34)	<0.0001
**CCI scores**					
0	437,037	53,522	0.31	(0.31–0.32)	<0.0001
≥1	393,486	105,134	0.30	(0.29–0.30)	<0.0001
**Coexisting comorbidities**					
Hypertension	437,193	112,774	0.33	(0.32–0.34)	<0.0001
Coronary artery disease	192,015	50,785	0.30	(0.29–0.31)	<0.0001
Stroke	123,990	58,964	0.34	(0.33–0.34)	<0.0001
Depression	56,147	13,352	0.32	(0.30–0.34)	<0.0001
Anxiety	117,630	24,118	0.33	(0.31–0.34)	<0.0001
Heart failure	57,194	27,547	0.32	(0.31–0.34)	<0.0001
Peripheral vascular disease	18,312	6472	0.34	(0.31–0.36)	<0.0001
COPD	175,048	56,398	0.31	(0.30–0.32)	<0.0001
Atrial fibrillation	18,823	10,415	0.35	(0.33–0.37)	<0.0001
Traumatic head injury	51,699	15,134	0.29	(0.27–0.30)	<0.0001
Hearing loss	22,724	6519	0.32	(0.30–0.35)	<0.0001
Sleep apnea	4772	840	0.33	(0.27–0.42)	<0.0001
Liver cirrhosis	237,795	46,407	0.29	(0.28–0.30)	<0.0001
SLE	13,139	2879	0.31	(0.27–0.34)	<0.0001
**DDD**					
≤1	560,998	137,268	0.36	(0.35–0.37)	<0.0001
>1	288,789	32,300	0.50	(0.46–0.53)	<0.0001
**Stain use**					
New use (after type 2 diabetes diagnosis)	803,889	159,321	0.31	(0.31–0.32)	<0.0001
Prevalent use (before type 2 diabetes diagnosis)	45,898	10,247	0.28	(0.26–0.29)	<0.0001
**Metformin use**	357,572	69,229	0.35	(0.34–0.36)	<0.0001

**Abbreviations**: ASMD, absolute standardized mean difference; SD, standard deviation; IQR, interquartile range; Q, quartile; DDD, defined daily dose; AIDS, acquired immunodeficiency syndrome; CCI, Charlson’s comorbidity index; COPD, chronic obstructive pulmonary disease; SLE, systemic lupus erythematosus; NTD, New Taiwan Dollar; aDCSI, adapted Diabetic Complication Severity Index; SU, sulfonylureas; AGI, alpha-glucosidase inhibitor; TZD, thiazolidinedione; DPP4i, dipeptidyl peptidase 4 inhibitor; SGLT2i, sodium–glucose cotransporter-2 inhibitor; aHR, adjusted hazard ratio; HR, hazard ratio; CI, confidence interval. * The aHR was derived from the inverse probability treatment-weighted Cox regression model considering statin use as a time-dependent covariate and was adjusted for age group, sex, income level, urbanization, antidiabetic drug type, antidiabetic drug use, aDCSI score, comorbidity, and CCI score.

## Data Availability

Data analyzed during the study were provided by a third party. Requests for data should be directed to the provider indicated in the Acknowledgments.

## Supplementary Materials

The following supporting information can be downloaded at: https://www.mdpi.com/article/10.3390/ph16040507/s1, Figure S1: Study flow-chart.
