# Supplementary figures and images for: Effects of Statin Dose, Class, and Use Intensity on All-Cause Mortality in Patients with Type 2 Diabetes Mellitus
Source: Pharmaceuticals (Basel). 2023 Mar 29;16(4):507. doi: 10.3390/ph16040507 (PMC10144141; doi:10.3390/ph16040507)

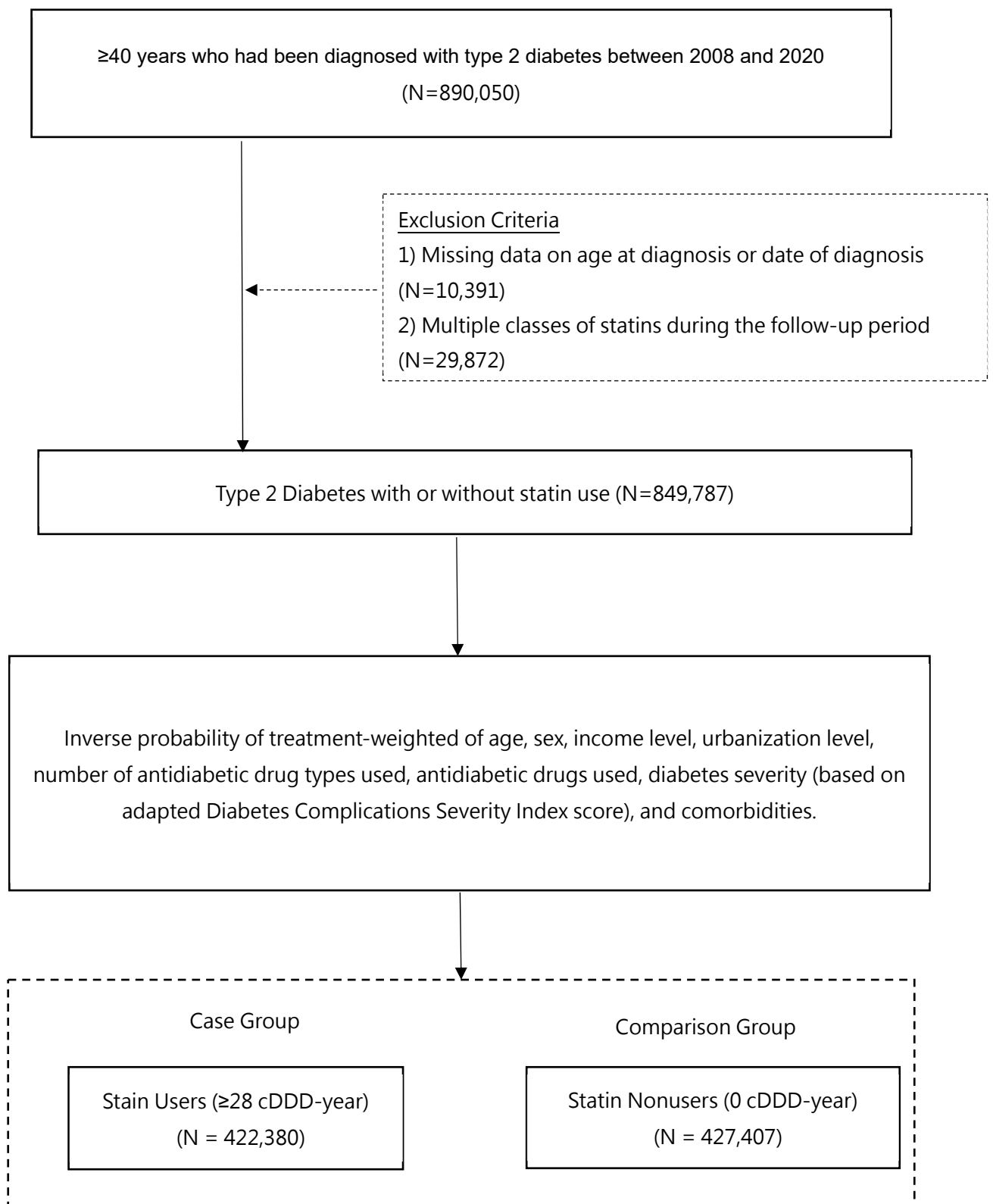

**Figure S1:** Study flow-chart

Supplement: Supplementary file 1 [file pharmaceuticals-16-00507-s001.zip › pharmaceuticals-2224136-supplementary.pdf]
